# A Rare Presentation of Infective Endocarditis Due to Serratia marcescens

**DOI:** 10.7759/cureus.22936

**Published:** 2022-03-07

**Authors:** Kajol Shah, Sarthak Patel, Sana Rashid, Meghana Subramanian, Victor Cueto

**Affiliations:** 1 Department of Internal Medicine, Rutgers University New Jersey Medical School, Newark, USA; 2 Medical School, Rutgers University New Jersey Medical School, Newark, USA; 3 Department of Internal Medicine and Pediatrics, Rutgers University New Jersey Medical School, Newark, USA

**Keywords:** fastidious, splenic abscess, serratia marcescens, septic arthritis, pneumonia, infective endocarditis, immunosuppression

## Abstract

*Serratia marcescens *is an opportunistic organism that can commonly cause respiratory tract infections in immunocompromised individuals. It has also been shown to cause urinary tract infections and soft tissue infections. It has several virulence factors including fimbriae-like adhesions that allow for surface attachment and biofilm formation to increase the likelihood of infections in humans. However, it has rarely been shown to cause infective endocarditis but has an increased mortality compared to the usual microbial agents associated with it (*Staphylococcus* and *Streptococcus*). Therefore, a high index of suspicion is necessary to accurately diagnose and treat patients at risk. Most published cases of *S. marcescens *endocarditis show that almost all described patients had chronic medical conditions or cardiovascular abnormalities. Furthermore, treatment has become difficult as* S. marcescens* has been shown to exhibit antibacterial resistance with beta-lactamase production.

Here, we present a complicated case of *S. marcescens *pneumonia and infective endocarditis with a good prognosis. Our patient had a rapid onset of complications (i.e. including joint infections, splenic abscesses, myositis, and septic arthritis), despite the initial benign presentation concerning for pneumonia. However, the patient had a favorable outcome due to the prompt work-up and treatment that was initiated. Therefore, *S. marcescens *bacteremia in a patient with risk factors should prompt further investigation with a thorough evaluation of source followed by immediate management. This case highlights the fastidious nature of *S. marcescens. *Further investigation needs to be done to elucidate the pathogenesis of the organism that can serve as a target for future therapeutic intervention.

## Introduction

*Serratia marcescens* is a facultative anaerobic, oxidase-negative, opportunistic, gram-negative nosocomial bacillus associated with intravenous drug use (IVDU), immunosuppression, previous antibiotic exposure, and indwelling catheter use [1-3]. It is found in various environmental settings like water, soil, and plants but is not part of the normal human flora [4-5]. It is commonly known to cause urinary tract infections, soft tissue infections, and respiratory tract infections and is the seventh most common cause of pneumonia, with an incidence rate of 4.1% [3]. It has multiple virulence factors such as fimbriae-like adhesions and biofilm formation, which allow for surface attachment and increase the likelihood of infections in humans [1]. It rarely is associated with a primary infection, but often operates as an opportunistic pathogen in immunocompromised hosts [3]. Those most at risk tend to be critically ill patients who are typically treated with broad-spectrum antibiotics [4].

Infective endocarditis is commonly caused by *Streptococcus* and *Staphylococcal* species and is associated with high mortality and morbidity [2]. Although rare, infection from gram-negative bacilli has increased in the recent years and is usually caused by HACEK (*Haemophilus, Aggregatibacter, Cardiobacterium, Eikenella*, and *Kingella* species) organisms. These cases of infective endocarditis have high in-hospital mortality. Non-HACEK infective endocarditis is rare with a 2% incidence and associated with poor outcomes [6]. *S. marcescens* endocarditis is extremely rare, accounting for only 0.14% of all cases of endocarditis [5,7]. Most published cases of it have been in patients who have chronic medical conditions or cardiovascular abnormalities [1]. Endocarditis due to it carries a high risk of mortality, especially in patients who received only non-surgical treatment where it is estimated to be as high as 85% [5]. The rarity of *Serratia* endocarditis and the high mortality risk associated with it warrant a high index of suspicion in order to promptly diagnose and treat susceptible patients. Here, we present a complicated case of *S. marcescens* pneumonia and infective endocarditis in a male intravenous drug user.

This case was presented at the virtual American Thoracic Society Conference in May 2021.

## Case presentation

The patient was a 50-year-old male with a past medical history notable for hepatitis C, IVDU, alcohol use disorder, hypertension, and an inguinal hernia, and presented to the emergency department in an altered mental state. He was brought in by Emergency Medical Services (EMS) because he was found wandering the streets after a fall. The patient reportedly did not suffer head trauma or loss of consciousness from the fall but endorsed having sharp chest pain, cough, and dyspnea on exertion. He also reported back pain and diffuse extremity pain. He denied having fevers, chills, orthopnea, nausea, vomiting, or urinary incontinence. Of note, the patient had a prior admission for alcohol intoxication to another hospital one month ago. He graduated from a rehab facility one year prior and was having minor relapses since then. He reported having last used intravenous heroin via a nonsterile needle three weeks prior to presentation and denied any recent cocaine, benzodiazepine, or alcohol use.

**Figure 1:**
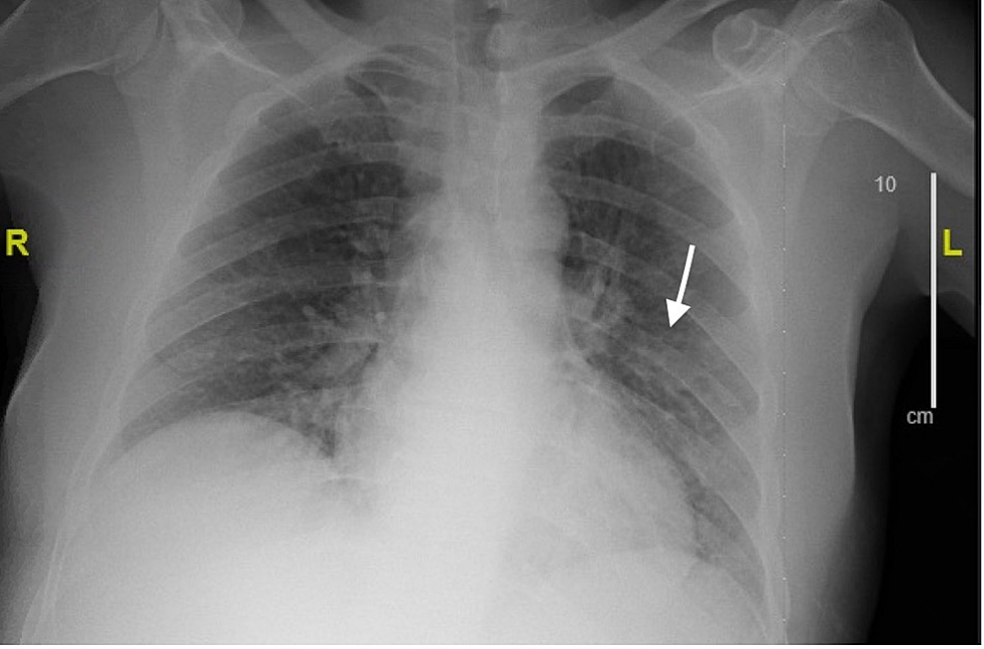
Chest X-ray significant of a left basilar opacity (arrow), most likely to represent consolidation, concerning for pneumonia

**Figure 2:**
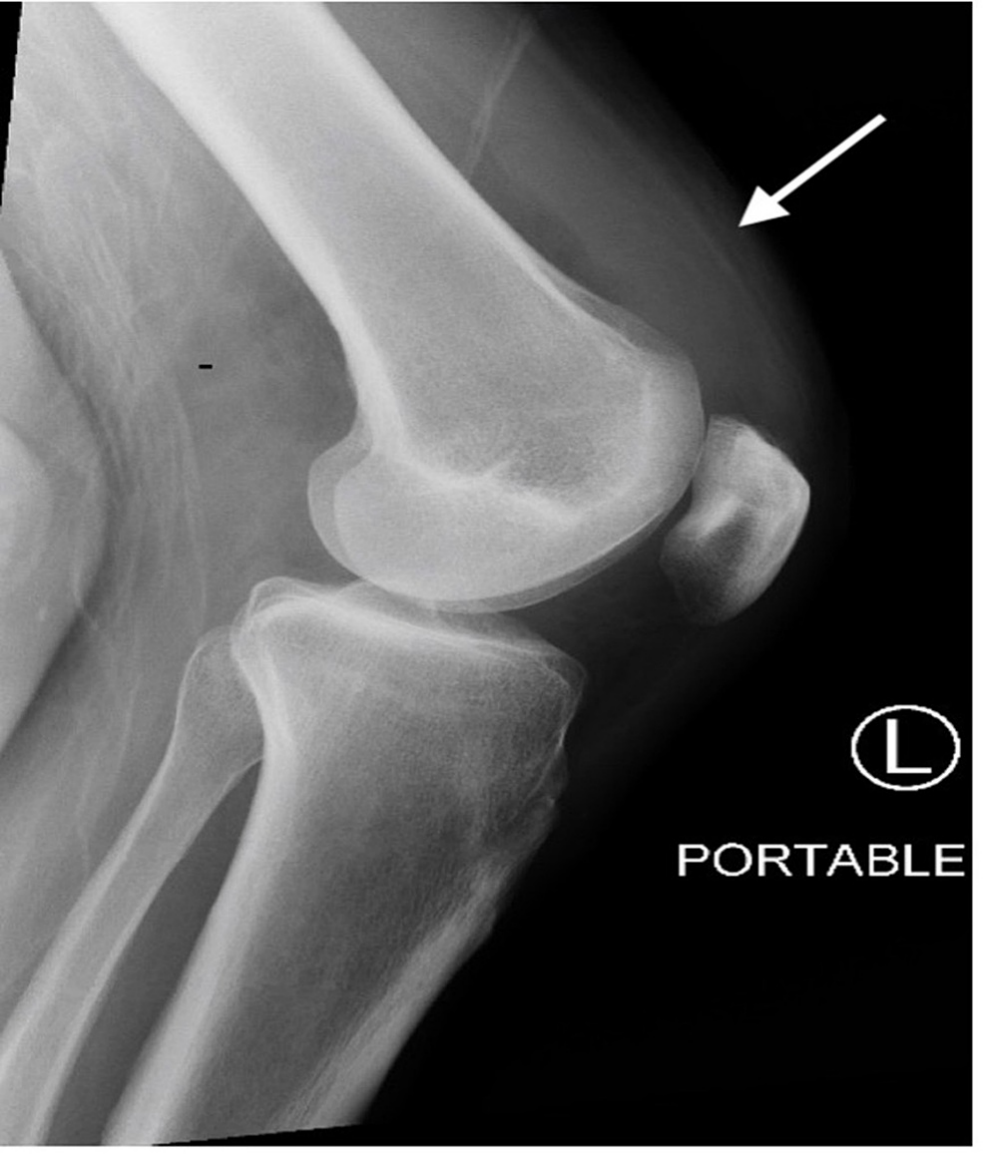
Lateral left knee X-ray revealing significant effusion (arrow), remarkable for septic arthritis

In the emergency department, vitals on presentation were as follows: temperature 99.1°F, blood pressure 157/60 mmHg, heart rate 87 beats per minute, respiratory rate 17 breaths per minute, and a SpO_2_ value of 100% on ambient air. On examination, the patient was oriented only to self and place, but not to time, being unable to tell what year it was. He was generally ill-appearing, lethargic, and diaphoretic. The exam was otherwise notable for decreased left basilar lung sounds, and generalized muscle tenderness in bilateral upper and lower extremities. Initial laboratory testing (Table 1) revealed a white blood cell count of 11.8 K/mL (reference 4.0-11.0 K/mL) with 90.1 % neutrophils (reference 35%-80%), platelets 25 K/mL (reference 150-450 K/mL), sodium 130 mEq/L (reference 133-145 mEq/L), potassium 3.0 mEq/L (reference 3.5-4.8 mEq/L), creatinine 1.6 mg/dL (reference 0.7-1.2 mg/dL), aspartate transaminase (AST) level of 172 U/L (reference 0-40 U/L), alanine transaminase (ALT) 56 U/L (reference 0-40 U/L), albumin 2.2 g/dL (reference 3.5-5.2 g/dL), total bilirubin 3.2 mg/dL (reference 0-1 mg/dL), creatinine kinase 2795 U/L (reference 0-200 U/L), troponin 0.21 ng/mL (reference 0.00-0.30 ng/mL), and urine drug screen positive for cannabis, opiates, and barbiturates. An EKG was significant for sinus tachycardia at a rate of 102 beats per minute, with no other abnormalities. Computed tomography (CT) of the head, cervical spine, and abdomen/pelvis was performed and was found to be unremarkable. Chest X-ray was significant for a left basilar opacity, concerning for pneumonia (Figure 1). His temperature spiked to 103.1°F while still in the emergency department. He was started on IV vancomycin 750 mg twice a day, IV piperacillin-tazobactam 4.5 g every six hours, and IV azithromycin 500 mg daily for a suspected left lower lobe pneumonia after collection of blood, urine, and sputum cultures. He was also started on intravenous fluids for an acute kidney injury and rhabdomyolysis. Thiamine, folate, vitamin B12, and multivitamin were also administered, and the patient was admitted to the medicine service for further management. The day after admission, the patient’s mental status began to improve. On the third day of admission, both the initial set of blood and urine cultures grew *S. marcescens.* Subsequent to this finding and antibiotic susceptibilities, the prior empiric antibiotics were discontinued, and the patient was started on IV meropenem 2 g every eight hours for extended-spectrum beta-lactamase (ESBL)-producing *Serratia*. Given the findings of positive blood cultures, a transthoracic echocardiogram was performed on the fourth day of admission, which showed no evidence of endocarditis. On the seventh day of admission, the patient began to report new-onset left knee swelling and pain, concerning for septic arthritis. A left knee X-ray was performed immediately and revealed a significant effusion concerning for septic arthritis (Figure 2). An arthrocentesis of the left knee yielded a synovial leukocyte count of 46,920 with 82% neutrophils, and the fluid was sent for culture, which ultimately grew *S. marcescens.*

**Table 1:** Initial laboratory findings

Laboratory test	Result	Reference value
White blood cell count	11.8 K/mL	4.0-11.0 K/mL
Platelets	25 K/mL	150-450 K/mL
Sodium	130 mEq/L	133-145 mEq/L
Potassium	3.0 mEq/L	3.5-4.8 mEq/L
Creatinine	1.6 mg/dL	0.7-1.2 mg/dL
Aspartate transaminase	172 U/L	0-50 U/L
Alanine transaminase	56 U/L	0-40 U/L
Albumin	2.2 g/dL	3.5-5.2 g/dL
Total bilirubin	3.2 mg/dL	0-1 mg/dL
Creatinine kinase	2795 U/L	0-200 U/L
Troponin	0.21 ng/mL	0.00-0.30 ng/mL

**Figure 3:**
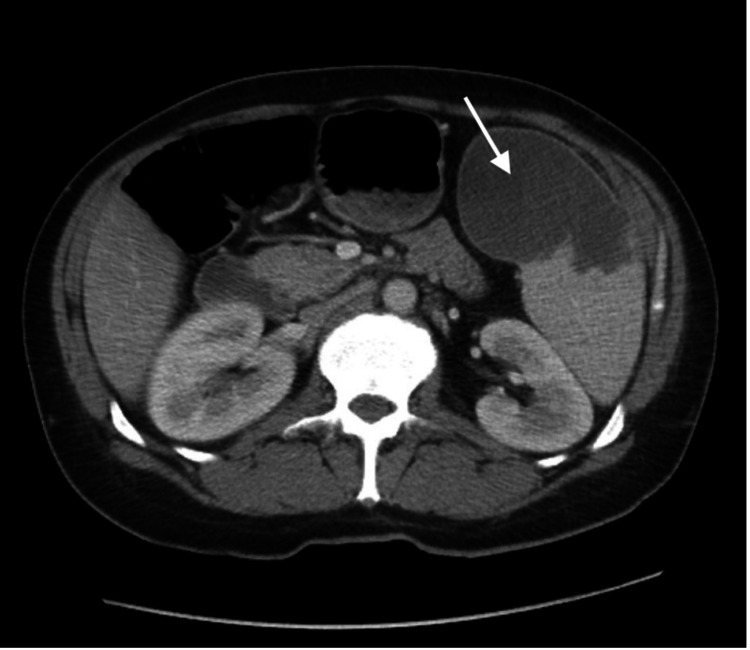
CT of the abdomen and pelvis demonstrating an enlarged spleen with an infarct in the inferior portion of the spleen (arrow)

**Figure 4:**
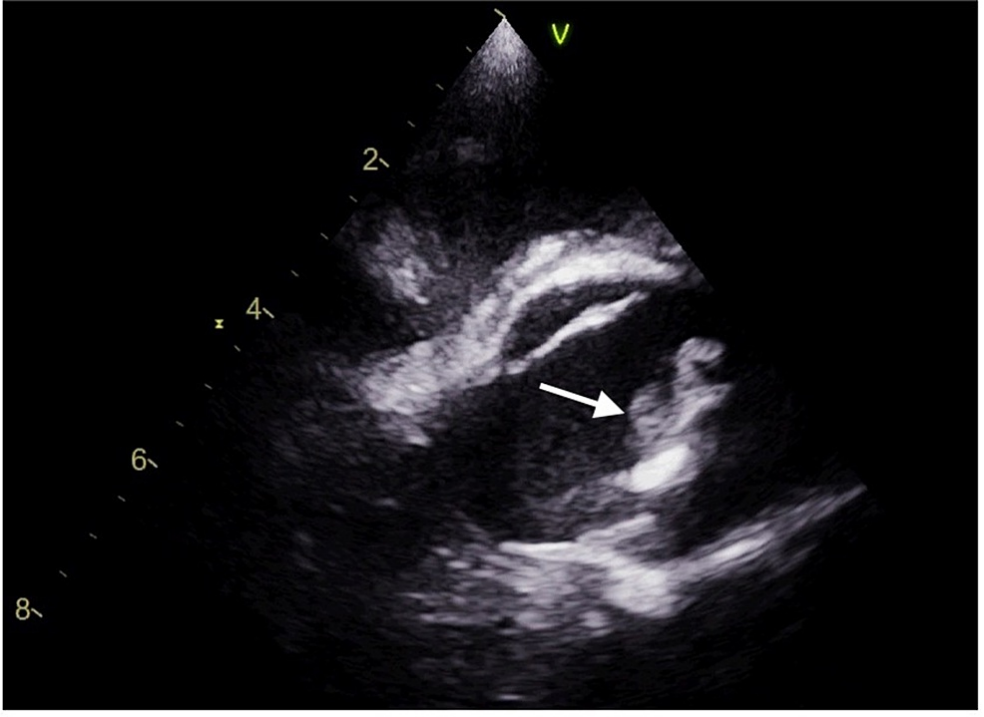
Parasternal long-axis view on transesophageal echocardiography revealing a large aortic valve vegetation (1.5 × 1.4 cm) and paravalvular abscess involving the aortic root

The patient continued to experience worsening back pain in the second week of hospitalization, prompting magnetic resonance imaging (MRI) of the lumbar spine to be done, which showed enhancement of the paraspinal musculature from L1-L4, suggestive of myositis. Follow-up CT of the abdomen done at the same time demonstrated small loculation with rim enhancement of an infarcted spleen (Figure 3). Interventional radiology consultation was done at this time. The fluid collection was drained, yielding 100 mL of serosanguinous fluid, and a drainage catheter was placed. Given the clinical presentation of multiple infectious foci, suspicion for endocarditis prompted performing a transesophageal echocardiogram that revealed a large aortic valve vegetation (1.5 × 1.4 cm) and paravalvular abscess involving the aortic root, with concomitant aortic regurgitation due to leaflet rupture (Figure 4). Cardiothoracic surgery consultation was made, and it was determined that the patient was not a good candidate for surgery at that time due to extensive damage of the valve as it needed time to heal. The patient was switched from meropenem to ertapenem 1 g daily for benefit of daily dosing, instead of thrice daily dosing. This was deemed as appropriate coverage of the septic foci at that time. His clinical status continued to improve significantly, including his mental status, with the patient regaining orientation to person, place, and time. He expressed an understanding for the need of sobriety and was seen by the addiction medicine service, with an appropriate follow-up arranged. The splenic drain was removed, and the patient was discharged after a month of hospitalization to a subacute rehabilitation facility, with a plan to continue intravenous ertapenem for a total duration of six weeks with a cardiothoracic surgery follow-up to plan definitive surgery of the aortic valve once the abscess resolved.

## Discussion

*S. marcescens* is an opportunistic bacterium that remains a rare cause of infective endocarditis and is most commonly described to cause nosocomial infections [1,8]. The main contributors to the development of disease are contamination and poor instrumentation technique [9-11]. Therefore, most patients are infected upon hospitalization, especially if they serve as a suitable compromised host [4]. Its progression to disease is not well understood and much still needs to be explored in terms of its clinical presentation and management [1]. Here, we present a case of what was initially thought to be community-acquired pneumonia but was later found to be *S. marcescens* endocarditis with multiple complications. Our patient did not have a known history of a recent iatrogenic manipulation that would have put him at risk of a *Serratia *infection; however, he was using nonsterile intravenous needles. Also, he did have a prior hospitalization for alcohol intoxication one month before this admission. His only initial sign of infection on presentation was leukocytosis, not meeting criteria for systemic inflammatory response syndrome (SIRS). Despite that, the bacteremia rapidly disseminated to multiple organ systems over the course of the next several days.

*S. marcescens* is a rare cause of pneumonia and has been reported in critically ill patients requiring prolonged ventilatory support [12]. In the outpatient setting, it has been associated with community-acquired pneumonia in those with a certain degree of immunocompromise [13]. In our patient, it is unlikely that this infection was the only cause of the rapid progression to the bacteremia witnessed later. The clinical course in this patient was notable for additional infectious foci (splenic abscesses, myositis, and septic arthritis), despite the initial presentation concerning for pneumonia. This infectious investigation was only initiated once both blood and urine cultures grew *S. marcescens* and the patient developed new symptoms of knee pain and worsening back pain. Given the first set of cultures returning positive, the patient likely already had disseminated infection upon presentation. Although there were no renal tract abnormalities seen on imaging, it is likely that the patient had an asymptomatic *Serratia* urinary tract infection that served as the primary source of bacteremia, or used a *Serratia-*contaminated needle. This highlights the insidious spread of the organism as the patient did not have any of the complications upon arrival at the emergency department. Therefore, *S. marcescens* bacteremia in a patient should immediately prompt further investigation with a thorough evaluation of the underlying source. Additionally, an intensified routine should be practiced for routine infection control measures once this bacterium is identified, as these measures have played a vital role in terminating outbreaks in the past [1]. Although recent reports have shown healthcare exposure as a common source of infection, historically, *S. marcescens* endocarditis has been shown to be associated with IVDU most notably in the San Francisco outbreak in the 1970s, where 89% of the 36 patients had a history of it [9-10]. Though most IVDU patients have a predilection of right-sided involvement of the heart, *S. marcescens* seems to prefer left-sided valves despite its strong association with IVDU [5]. Left-sided native valve endocarditis has been shown to have an increased rate of mortality compared to right-sided lesions, due to a higher incidence of complications and increased rate of systemic embolization [14]. This likely explains the higher rate of paravalvular complications and valvular destructions that are seen with *S. marcescens* endocarditis [5]. Our patient already had a paravalvular abscess and aortic valve leaflet rupture at the time the transesophageal echocardiogram was performed. This highlights the severity and complex nature of *S. marcescens *endocarditis. Given that *S. marcescens* endocarditis is associated with high morbidity and mortality, a high suspicion should be held for a bacteremia especially in patients who have the epidemiological risk factors that include IVDU, immunosuppression, and frequent hospitalizations.

*S. marcescens* endocarditis does not have definitive treatment guidelines due to its rarity. Furthermore, its known history of multi-drug resistance to different classes of antibiotics makes it challenging to treat [11]. Therefore, resistant strains are one of the hallmarks of reported *S. marcescens* outbreaks in the past and the treatment approach has been heterogeneous as seen in this case [1]. Gram-negative infective endocarditis is typically treated with an aminoglycoside (e.g. IV gentamicin) or a third-generation cephalosporin [6]. Both of these choices were given consideration in our case before culture sensitivities returned, but due to the rapid onset of septic complications, antibiotics were escalated to meropenem. The 2015 Infectious Diseases Society of America (IDSA) guidelines recommend combination therapy with a beta-lactam and an aminoglycoside or fluoroquinolone for six weeks for *S. marcescens* endocarditis [15]. Other antibiotics that *S. marcescens* has been shown to be susceptible to are third- and fourth-generation cephalosporins, carbapenems, and monobactams [5]. However, culture sensitivities should also be used to guide therapy. In the setting of increased risk of blood-borne infections, it is crucial to recognize less-frequent causes of infective endocarditis and promptly initiate an infectious workup and treatment. Here, we immediately escalated antibiotics to a carbapenem once cultures began growing gram-negative bacteria that later resulted in ESBL-producing *S. marcescens*. Furthermore, considering treatment with only medical therapy is more successful in right-side endocarditis, surgical consultation should be made preferably within the first 7-10 days [9,16]. Early surgical treatment aims to reduce embolic events [17]. Surgical consultation was immediately made but due to the extensive damage and location of the lesion, the patient was treated medically with a good outcome.

## Conclusions

Infectious endocarditis with *S. marcescens* is rare and may not be evident on initial presentation, as illustrated in this case. Here, we have presented a rare case of *S. marcescens* infectious endocarditis with aortic involvement and septic emboli. The case also highlights the pathogenicity of *S. marcescens*, with rapid spread of infection to multiple organs. Further investigation needs to be done to determine virulence factors that may contribute to the pathogenesis. This can help prevent future outbreaks especially in the immunosuppressed population. Considering there are no set guidelines for treatment, our case highlights the importance of early suspicion and therapeutic intervention that resulted in a favorable outcome here and can guide future cases.

## References

[1] Phadke VK, Jacob JT (2016). Marvelous but morbid: infective endocarditis due to Serratia marcescens. Infect Dis Clin Pract (Baltim Md).

[2] Guler S, Sokmen A, Mese B, Bozoglan O (2013). Infective endocarditis developing serious multiple complications. BMJ Case Rep.

[3] (2020). Serratia marcescens. http://www.antimicrobe.org/b26.asp.

[4] Khanna A, Khanna M, Aggarwal A (2013). Serratia marcescens - a rare opportunistic nosocomial pathogen and measures to limit its spread in hospitalized patients. J Clin Diagn Res.

[5] Yeung HM, Chavarria B, Shahsavari D (2018). A complicated case of Serratia marcescens infective endocarditis in the era of the current opioid epidemic. Case Rep Infect Dis.

[6] Falcone M, Tiseo G, Durante-Mangoni E (2018). Risk factors and outcomes of endocarditis due to non-HACEK gram-negative bacilli: data from the prospective multicenter Italian endocarditis study cohort. Antimicrob Agents Chemother.

[7] Richardson A, Martinez A, Ghetiya S, Missov E, Percy R, Sattiraju S (2020). Serratia marcescens endocarditis with perivalvular abscess presenting as atrioventricular block. Case Rep Infect Dis.

[8] Morpeth S, Murdoch D, Cabell CH (2007). Non-HACEK gram-negative bacillus endocarditis. Ann Intern Med.

[9] Elkattawy S, Mohammadian M, Williams N (2021). Serratia marcescens endocarditis. Cureus.

[10] Mills J, Drew D (1976). Serratia marcescens endocarditis: a regional illness associated with intravenous drug abuse. Ann Intern Med.

[11] Mahlen SD (2011). Serratia infections: from military experiments to current practice. Clin Microbiol Rev.

[12] Carlon GC, Dickinson PC, Goldiner PL, Turnbull AD, Howland WS (1977). Serratia marcescens pneumonia. Arch Surg.

[13] Zarogoulidis P, Porpodis K, Konoglou M (2011). Serratia pneumonia presenting as hemoptysis in a patient with sarcoidosis: a case report. Int J Gen Med.

[14] Kamaledeen A, Young C, Attia RQ (2012). What are the differences in outcomes between right-sided active infective endocarditis with and without left-sided infection?. Interact Cardiovasc Thorac Surg.

[15] Baddour LM, Wilson WR, Bayer AS (2015). Infective endocarditis in adults: diagnosis, antimicrobial therapy, and management of complications: a scientific statement for healthcare professionals from the American Heart Association. Circulation.

[16] Hadano Y, Kamiya T, Uenishi N (2012). A fatal case of infective endocarditis caused by an unusual suspect: Serratia marcescens. Intern Med.

[17] Leblebicioglu H, Yilmaz H, Tasova Y (2006). Characteristics and analysis of risk factors for mortality in infective endocarditis. Eur J Epidemiol.

